# Iatrogenic diaphragmatic herniation of the liver into the pericardium—a case report

**DOI:** 10.1093/jscr/rjad456

**Published:** 2023-08-14

**Authors:** Phoebe C Wood, Fraser H Simpson, Peter Yuide, Robert Franz

**Affiliations:** Department of General Surgery, The Prince Charles Hospital, Chermside, Australia; Department of General Surgery, The Prince Charles Hospital, Chermside, Australia; Northside Clinical School, University of Queensland, Brisbane, Australia; Department of General Surgery, The Prince Charles Hospital, Chermside, Australia; Department of General Surgery, The Prince Charles Hospital, Chermside, Australia

**Keywords:** General Surgery, Hernia, Liver, Pericardium, Transdiaphragmatic

## Abstract

Iatrogenic diaphragmatic herniation is rare. This case is an example of herniation of the liver into the pericardial space post-transdiaphragmatic pericardial window formation for recurrent pericarditis. This case highlights that transdiaphragmatic herniation of intra-abdominal organs should be considered in patients presenting with gastrointestinal or cardiorespiratory symptoms with history of iatrogenic diaphragmatic defect.

## INTRODUCTION

Herniation of intra-abdominal organs through iatrogenic diaphragmatic defects is a rare phenomenon and can result in significant complications secondary to strangulation of herniated organs or associated cardiorespiratory compromise. Non-specific gastrointestinal and cardiorespiratory symptoms can also prolong diagnosis and definitive management.

Formation of a transdiaphragmatic pericardial window, first described in 1992 [1], has been identified as a simple and safe approach to management of recurrent pericardial effusion [2]. This approach eliminates the need for indwelling chest-drains and single-lung ventilation [3]. However, the creation of communication between the pericardial and peritoneal cavities can result in an iatrogenic intrapericardial hernia.

## CASE REPORT

A 38-year-old female presented to the emergency department with severe, pleuritic left sided pain. Her symptoms were thought to be secondary to a flare of recurrent idiopathic pericarditis secondary to recurrent serositis for which she had undergone a laparoscopic transdiaphragmatic pericardial window formation 7 months prior. This procedure was uncomplicated and involved the creation of a 3 mm window between the peritoneal and pericardial spaces.

**Figure 1:**
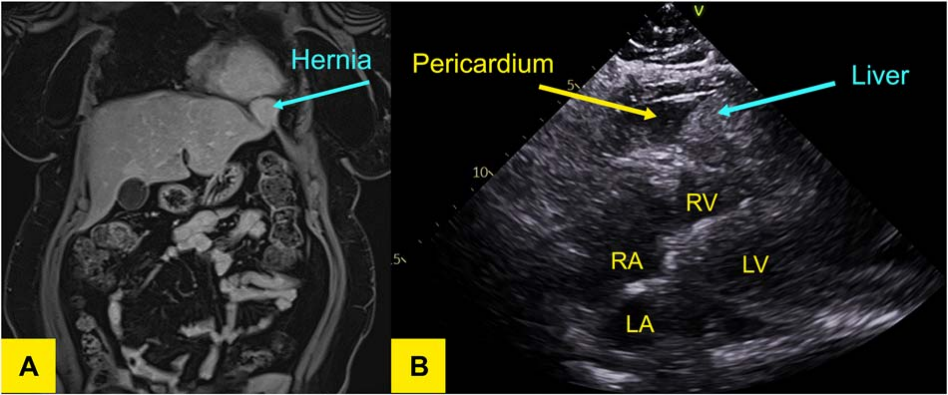
(**A**) T1-weighted MR of the liver in coronal plane showing a wide neck hernia at the anterolateral aspect of left hemidiaphragm that contains liver tissue and bulges outside the liver contour. No signal/enhancement abnormality. (**B**) Transthoracic echocardiogram demonstrating liver in pericardium adjacent to left ventricle.

An echocardiogram revealed moderate loculated pericardial effusion and an echo dense mass within the pericardium measuring 4.2 × 4.3 cm creating mass effect on the right ventricular free wall. The defect was better appreciated on a subsequent MRI of the liver (Fig. 1A).

**Figure 2:**
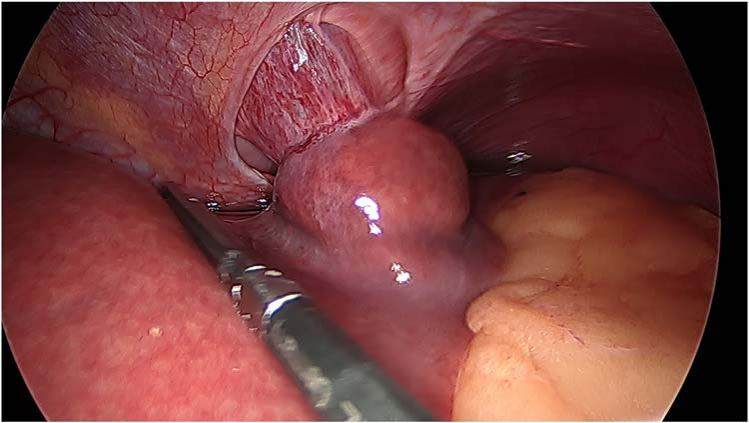
Intraoperative photo of portion of Segment 2 of the liver herniating through defect in central tendon of diaphragm and into pericardial sac. Pericardial adhesions are connected to the capsule of liver.

Laparoscopy was performed that revealed herniation of Segment 2 of the liver through a defect at the site of previous window formation (Fig. 2). Adhesiolysis of tissue attaching the liver to the diaphragm was performed and the plug of liver freed. There was no macroscopic evidence of vascular compromise to the herniated portion of liver.

**Figure 3:**
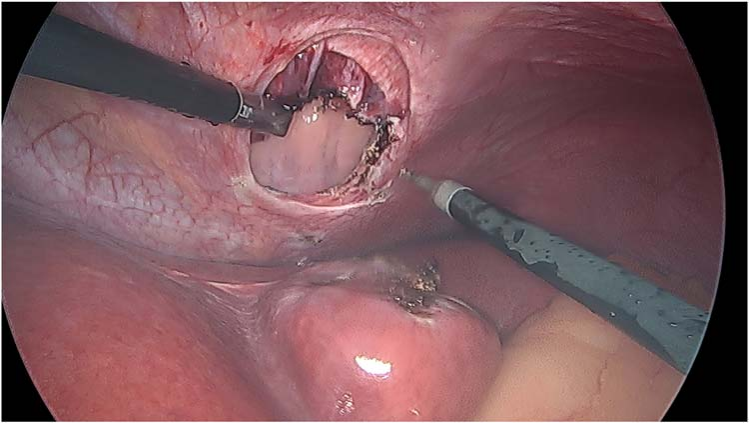
Intraoperative photo demonstrating the defect in the central tendon of the diaphragm into the pericardium after the liver had been reduced. The liver appeared viable on inspection.

**Figure 4:**
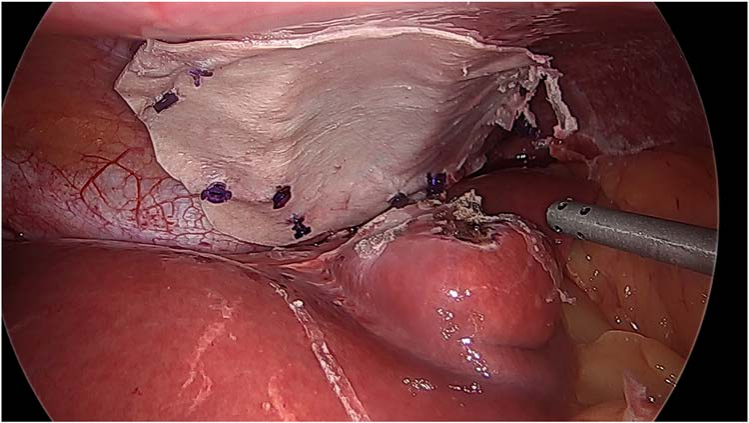
Intraoperative photo of the repair with bovine pericardial tissue.

There was an obvious 2 × 2 cm defect in the diaphragm allowing for communication with the pericardial sac. The inferior aspect of the heart was visible through this defect (Fig. 3). Bovine pericardial tissue was glued using Glubran 2® and Absorbatak® to the inferior aspect of the diaphragm to cover the defect. Glubran 2® was applied intermittently around the circumference of the patch, preventing a complete seal from being created and continuing to allow excess pericardial fluid to drain into the peritoneal space (Fig. 4).

The patient has since been reviewed in general surgical clinic. She has not had any further presentations to the emergency department for pericardial effusion or complications post-repair procedure.

## DISCUSSION

Whilst pericardial herniation is more commonly associated with trauma or congenital defect, iatrogenic herniation is a novel presentation with only a handful of previously documented cases. Several of these have described symptoms of strangulation or obstruction of bowel [4, 5], whereas others have described the mass effect of herniation on cardiovascular and respiratory function resulting in dyspnoea and chest discomfort [4, 6–9]. In this case, the patient’s previous symptoms were mimicked by irritation of the pericardium by the herniation liver.

Conditions in which intra-abdominal pressure is increased, such as in chronic cough, have been identified as possible risk factors for herniation post-diaphragmatic pericardial window formation [5]. At time of re-operation many of the diaphragmatic defects had increased in size increasing risk of herniation [6, 10]. The formation of smaller pericardial windows has been suggested to allow for this natural expansion [10]; however, as this case demonstrates, with only a 3 mm defect being created, it does not eliminate the risk of expansion and subsequent herniation.

Patients should be adequately counselled on the risks of potential abdominal organ herniation prior to laparoscopic pericardial window formation. Despite its novelty transdiaphragmatic hernia should be considered as a differential for patients presenting with gastrointestinal or cardiovascular symptoms who have previously undergone transdiaphragmatic pericardial window formation.
